# CEST theranostics: label-free MR imaging of anticancer drugs

**DOI:** 10.18632/oncotarget.7141

**Published:** 2016-02-02

**Authors:** Yuguo Li, Hanwei Chen, Jiadi Xu, Nirbhay N. Yadav, Kannie W. Y. Chan, Liangping Luo, Michael T. McMahon, Bert Vogelstein, Peter C.M. van Zijl, Shibin Zhou, Guanshu Liu

**Affiliations:** ^1^ F.M. Kirby Research Center for Functional Brain Imaging, Kennedy Krieger Institute, Baltimore, Maryland, USA; ^2^ The Russell H. Morgan Department of Radiology and Radiological Science, Division of MR Research, Johns Hopkins University School of Medicine, Baltimore, Maryland, USA; ^3^ Department of Radiology, Panyu Central Hospital, Guangzhou, China; ^4^ Department of Radiology, The First Affiliated Hospital of Jinan University, Guangzhou, China; ^5^ Ludwig Center, Howard Hughes Medical Institute and Sidney Kimmel Cancer Center, Johns Hopkins University School of Medicine, Baltimore, Maryland, USA

**Keywords:** CEST, MRI, theranostics, image-guided drug delivery, chemotherapy

## Abstract

Image-guided drug delivery is of great clinical interest. Here, we explored a direct way, namely CEST theranostics, to detect diamagnetic anticancer drugs simply through their inherent Chemical Exchange Saturation Transfer (CEST) MRI signal, and demonstrated its application in image-guided drug delivery of nanoparticulate chemotherapeutics. We first screened 22 chemotherapeutic agents and characterized the CEST properties of representative agents and natural analogs in three major categories, i.e., pyrimidine analogs, purine analogs, and antifolates, with respect to chemical structures. Utilizing the inherent CEST MRI signal of gemcitabine, a widely used anticancer drug, the tumor uptake of the i.v.-injected, drug-loaded liposomes was successfully detected in CT26 mouse tumors. Such label-free CEST MRI theranostics provides a new imaging means, potentially with an immediate clinical impact, to monitor the drug delivery in cancer.

## INTRODUCTION

Cancer still remains one of the most formidable diseases to cure. Currently, curing unresectable cancers mainly relies on chemotherapies, but the clinical outcome is discouraging, and the patients’ quality of life is often poor due to the severe adverse effects. Achieving effective anticancer drug therapy requires not only a certain level of effectiveness of an anticancer drug against specific types of cancer cells, but also the ability to deliver enough of the drug to exceed a threshold effective level of activity over the full anatomic extent of the cancer cell population. The heterogeneity of the tumor often results in unpredictable outcomes in individual patients [1, 2]. Thus, it is essential to develop tools with which to assess whether drugs are delivered to the tumor at an adequate concentration and subsequently adjust the treatment plan accordingly, a so-called “personalized medicine” strategy [3], in which non-invasive imaging modalities are expected to play a central role. Currently, there is an extensive investment in the development of molecular imaging techniques that can assess the effectiveness of drug delivery to the tumor [4]. One technical hurdle for the implementation of these approaches, however, is the requirement to chemically or physically attach imaging probes to the drug molecules or drug carriers, which may hamper clinical translation.

**Figure 1:**
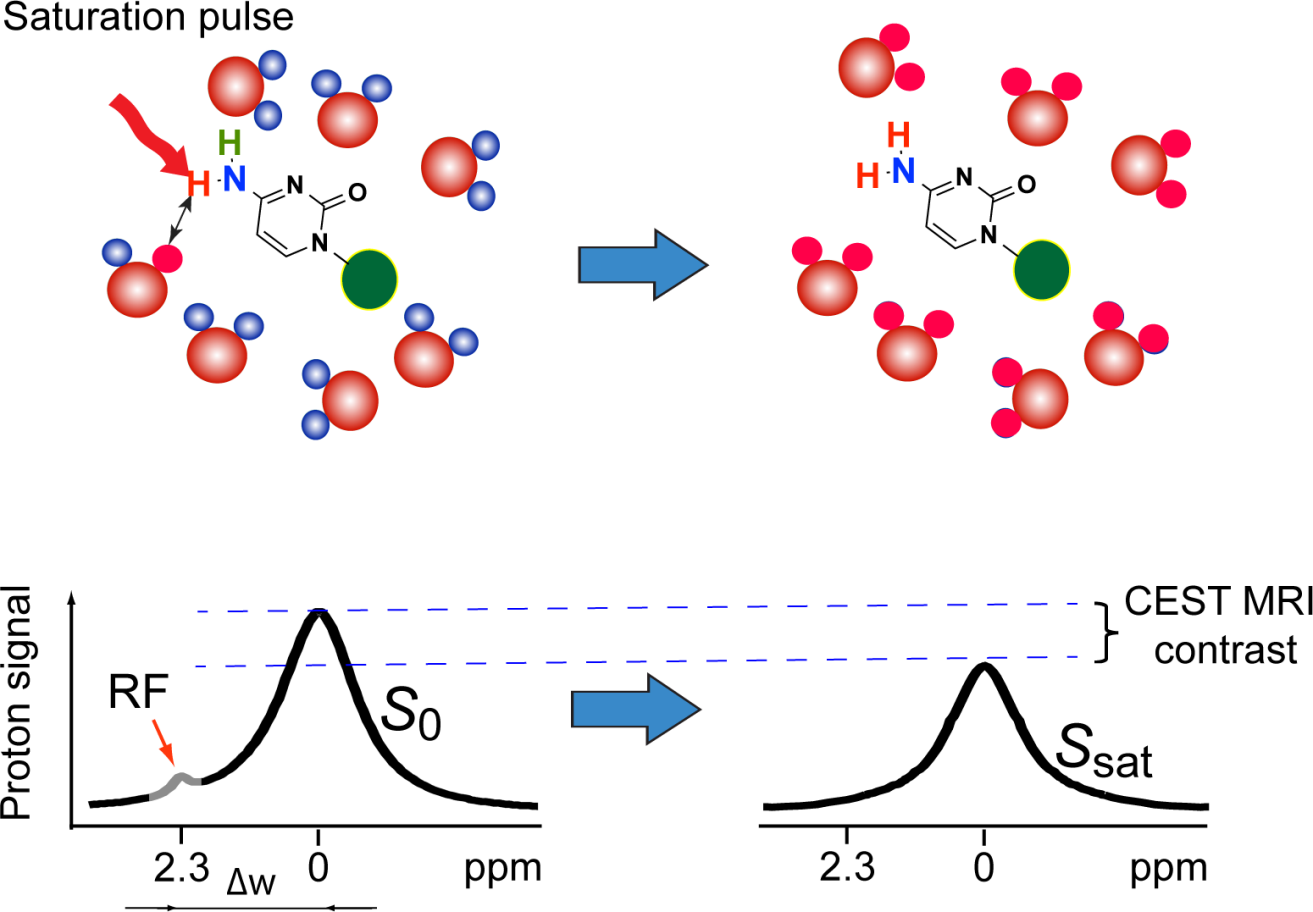
The principle of CEST MRI detection of anticancer drugs, such as gemcitabine Exchangeable protons on the drug molecules can transfer RF saturation to the protons of surrounding water (top row), resulting in a decrease in MRI signal. Continuously applying RF pulses leads to the saturation of more water protons, generating a detectable MRI contrast called Chemical Exchange Saturation Transfer (CEST) contrast (bottom row).

In this present study, Chemical Exchange Saturation Transfer (CEST) MRI [5-7] is utilized to directly detect non-chemically labeled chemotherapeutic agents. CEST contrast agents, unlike the commonly used T1 and T2 contrast agents, do not rely on the use of paramagnetic labels (i.e., Gd, Fe, or Mn), which makes the use of highly biocompatible, diamagnetic compounds possible. As shown in Figure 1, CEST MRI contrast is generated by the continuous application of RF saturation pulses at the resonance of exchangeable protons in the CEST agent and results in saturated protons (protons with nulled NMR signal), which, due to the nature of the proton exchange, are continuously transferred to the surrounding water, resulting in the saturation of a significant portion of the water MR signal. Practically, the requirement for a diamagnetic compound to be CEST MRI-detectable is to have water-exchangeable protons with a slow-to-moderate exchange rate (i.e., k_ex_ <<Δω, where k_ex_ is the exchange rate and Δω is the frequency difference between the chemical shifts of a given exchangeable proton and the water protons) and an offset sufficiently far from the water proton resonance to avoid interference of large direct saturation effects, which are often the case for a wide array of medically relevant compounds, including glucose [8, 9], glutamate [10] and peptides [11], proteins [12], pyrimidine compounds [13] and even therapeutic bacterial cells [14]. Based on our previous studies on the relationship between CEST properties and chemical structures, we hypothesized that CEST MRI could be used for the label-free detection of many anticancer drugs using their inherent exchangeable protons, in hydroxyl (OH), amide(NH), and amine (NH_2_) groups.

## RESULTS AND DISCUSSION

### Cytidine analogue anticancer drugs can be directly detected by CEST MRI

**Figure 2:**
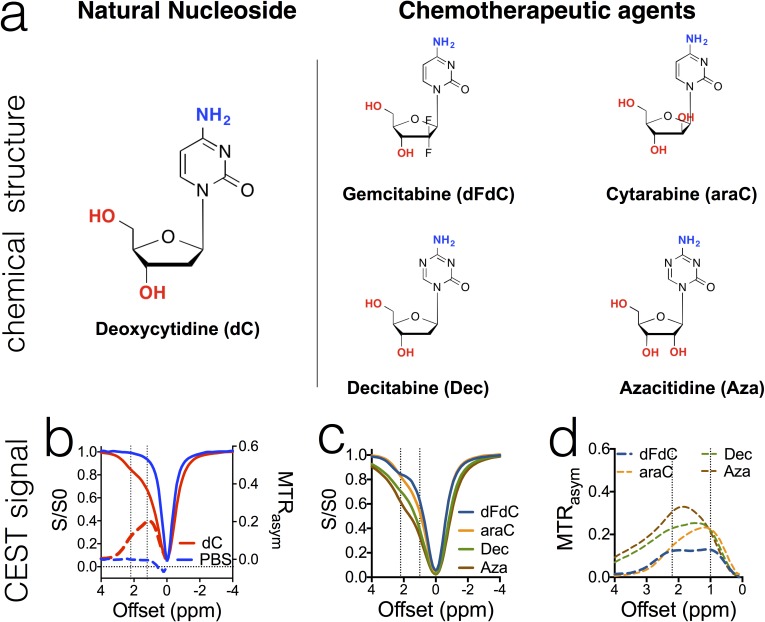
The chemical structure of cytidine- based agents (a) and their CEST MRI contrast, as shown both by z-spectra (b & c) and MTR^asym^ plots (b & d) All samples were prepared in PBS (pH 7.4) at a concentration of 20 mM and measured at 37°C using a 3.6 μT, 3 sec CW RF pulse.

To test our hypothesis, we first examined the four widely used cytidine analogue anticancer drugs, gemcitabine (dFdC), cytarabine (araC), decitabine (Dec), and azacitidine (Aza), which are either approved or in clinical trials, and their natural analog deoxycytidine (dC). As shown in Figure 2a, all drugs have a chemical structure similar to that of deoxycytidine. Our *in vitro* results showed that, as expected, all of these anticancer drugs (in PBS solution, pH = 7.4 and 37°C) exhibited two strong CEST MRI signals, around 2.0-2.4 ppm and 1.0 ppm, corresponding to the amino and hydroxyl protons respectively. In Figure 2b-2d, CEST signals are shown in both Z-spectra (solid lines), in which the water proton signal is plotted as a function of saturation frequency, and in MTR_asym_ plots (dotted lines), a more quantitative metric defined by: MTR_asym_ = (S^−Δω^ - S^Δω^)/S_0_, where S^−Δω^ and S^Δω^ are the MRI signal intensities after saturation at negative and positive values of the offset frequency Δω from the water proton frequency (set at 0 ppm by convention); S_0_ is the intensity in the absence of a saturation pulse. Maximal MTR_asym_ values of 0.119±0.007 and 0.129±0.025 could be achieved for 20 mM dFdC at offsets of 2.2-2.3 ppm and +1.0 ppm, respectively, when a continuous wave RF (B_1_ = 3.6 μT and t_sat_ = 4 sec) saturation pulse was used.

### The pH dependence of CEST MRI signal of cytidine-based anticancer drugs

**Figure 3:**
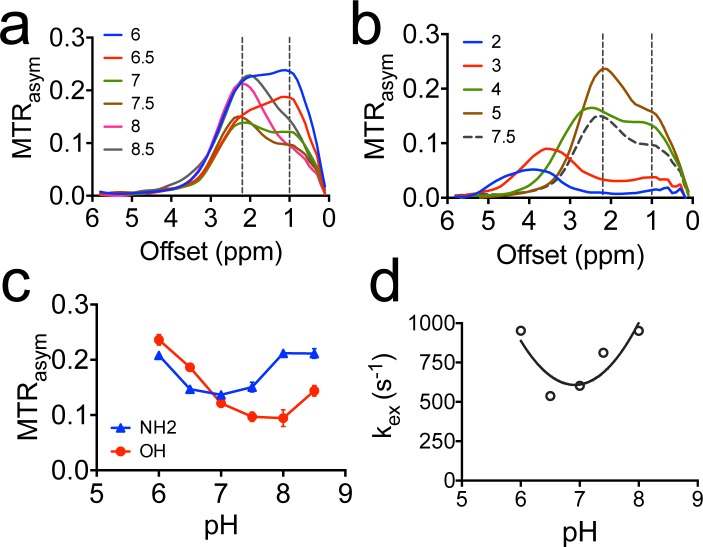
The pH dependence of CEST contrast of dFdC **a.** The MTR_asym_ plots of 20 mM dFdC in the pH range from 6 to 8.5; **b.** The MTR_asym_ plots of 20 mM dFdC in a pH range from 2 to 5. The pH 7.5 is also plotted as a reference; **c.** The pH dependence of CEST contrast of amine and hydroxyl in a pH range from 6.0 to 8.5; **d.** The pH dependence of the exchange rate of NH2 in a pH range from 6.0 to 8.0 using the FLEX method.

We measured the CEST contrast of gemcitabine in a pH range from 2 to 9, as shown in Figure 3. We also used the frequency-labeled exchange (FLEX) transfer method as previously described [13, 15] to determine the exchange rate of the amine protons of dFdC at different pH. The results revealed a strong pH effect on the CEST contrast of hydroxyl protons. For example, the CEST MRI signal of OH protons increases dramatically (> 2 times) when the pH drops from pH 8 to pH 6. In contrast, the CEST contrast of NH_2_ is relatively stable in pH range from 6.5 to 7.5. For this reason, we chose the CEST MRI signal of NH_2_ (i.e., ∼2.3 ppm in the pH range of 6.5 to 7.5) for quantifying dFdC. Similar pH dependences for other drugs were observed (Figure S2).

Interestingly, at pH 3, the CEST contrast of OH is completely gone while that of NH_2_ is shifted from 2.2 ppm to 3.4 ppm (Figure 3b). The pKa of the amine protons of dFdC was estimated to be 4.3 (Table S1, calculated using Advanced Chemistry Development (ACD/Labs) Software V11.02). Hence, the shift of amine CEST is likely due to the protonation of NH_2_ (pKa = 4.3) and the protonated NH^3+^ having a new chemical shift at ∼ 3.4 ppm. However, it may stem from the OH protons of sugar [9]. The exact assignment of this chemical shift is still under investigation.

### Sensitivity of the CEST MRI detection

**Figure 4:**
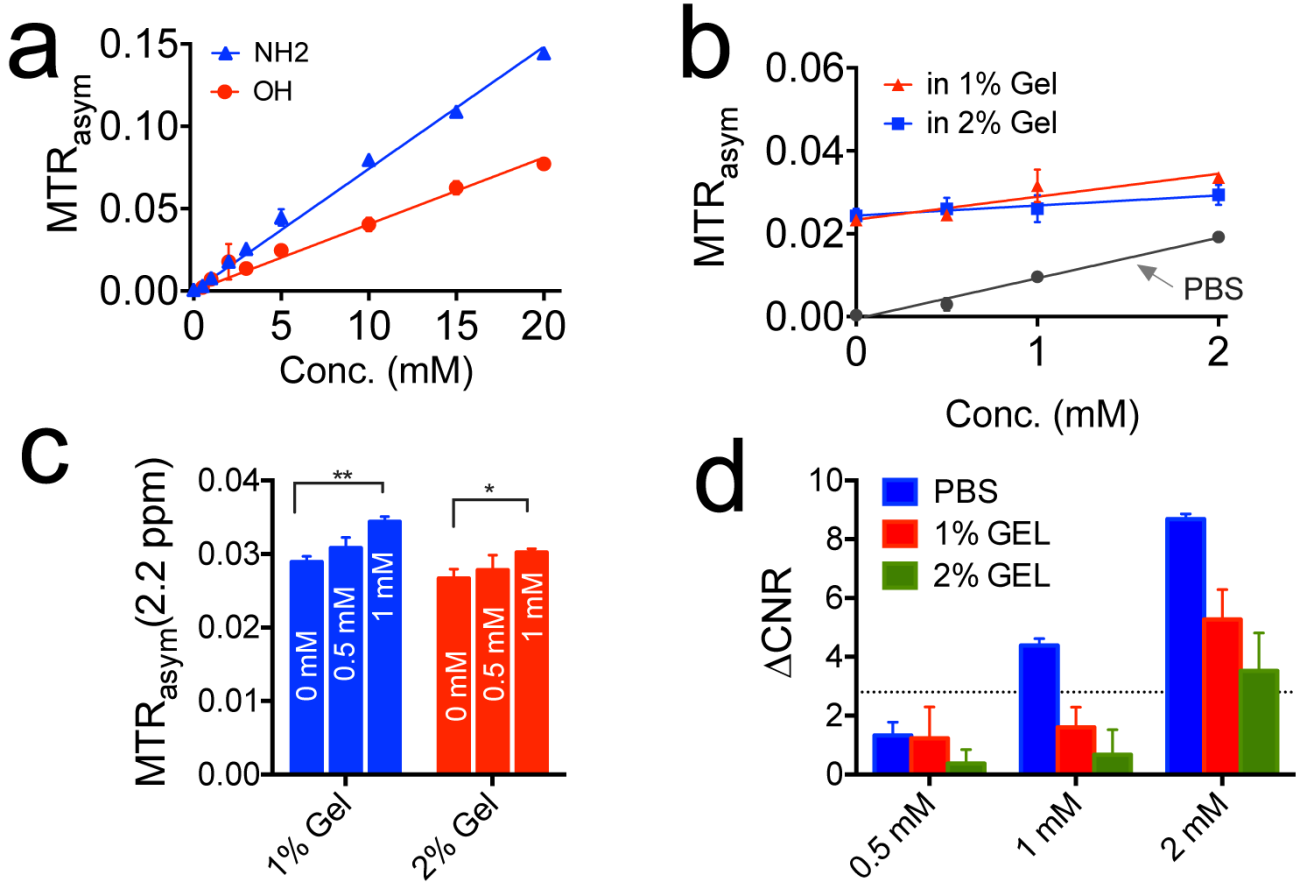
The detection limit of CEST MRI for detecting gemcitabine **a.** The calibration curves of the CEST signal of gemcitabine at concentrations ranging from 0.5 to 20 mM in PBS; **b.** The CEST MRI contrast at 2.2 ppm for samples prepared in 1% and 2% agarose gels, the CEST contrast in PBS is shown for comparison; **c.** Bar plots of the CEST contrast at different concentrations in each sample. A two-tailed paired Student's t-test was performed (**p < 0.01 and *P < 0.05). The results are mean ± SD (*n* = 3); **d.** Bar plots of the ΔCNR for samples containing gemcitabine at different concentrations as compared to reference samples containing 1% or 2% agarose gel. The horizontal dashed line represents ΔCNR *=* 2√2.

To evaluate the minimal concentration for using CEST MRI to detect gemcitabine, we performed CEST MRI on the samples containing gemcitabine at a concentration ranging from 0.5 mM to 20 mM. To mimic the *in vivo* baseline magnetization transfer effect, we also prepared samples in 1% or 2% agarose gel. The results are shown in Figure 4. Figure 4a shows that the MTR_asym_ values at 2.3 ppm and 1.0 ppm, for NH_2_ and OH protons respectively, have a very good linearity in the concentration below 10 mM, indicating that the MTR_asym_ can be used directly to quantify concentration. Figure 4b shows the CEST signal of NH_2_ protons of dFdC at low concentrations (i.e., 0.5 to 2 mM) in 1% and 2% agarose gel phantoms. Interestingly, agarose gel itself has a small but detectable CEST signal, suggesting it may be a better phantom to determine the minimum detection sensitivity by mimicking the *in vivo* condition.

We used two methods to determine the minimum detection sensitivity. First, we performed a statistical analysis (the two-tailed, unpaired Student's *t* test, *n* = 3) to determine the minimum concentration of gemcitabine that can generate a significant difference in CEST signal between just agarose gel and gemcitabine containing agarose gel. As shown in Figure 4c, a significant difference could be achieved for samples containing 1 mM or higher gemcitabine but not those containing 0.5 mM gemcitabine. The P values were determined to be 0.0007 and 0.0106 for 1 mM gemcitabine in 1% and 2% agarose gel respectively, with both showing significant differences (i.e., *P* < 0.05) compared to the reference gel samples. Second, we also adapted a previously published contrast-to-noise Ratio (CNR)-based approach [16] to determine the detection threshold in the presence of systemic noise. As shown in Figure 4d, when a ΔCNR threshold of 2√2 was used [16], the minimum detection sensitivity was estimated to be <1 mM for PBS samples and 1-2 mM for gel samples. It should be noted that the estimation of CEST detectability is affected by the choice of CNR threshold and CNR thresholds in different range (i.e., 0.6-2) have been reported previously [17, 18]. When a ΔCNR threshold = 1 was used, for instance, the detection limit was estimated to be 0.5 mM for gemcitabine in 1% agarose gel. In comparison, the typical detectability for small molecular Gd-based contrast agents is in the range of hundreds μM to mM [19] and that of ^19^F MRI is in the range of tens of mM [20]. Our study suggests that CEST MRI can provide a similar detectability as those imaging contrast generation strategies.

It also should be noted that the results shown in Figure 4 were acquired using a 3.6 μT, 3-second CW saturation pulse at a spatial resolution of 130×130 μm^2^. Because the saturation parameters significantly affect the MTR_asym_, a new, saturation-parameter-independent metric for quantifying CEST contrast may be more useful to determine the detectability (or sensitivity) of a CEST agent.

### CEST MRI can be used to detect antitumor drugs in other categories

**Figure 5:**
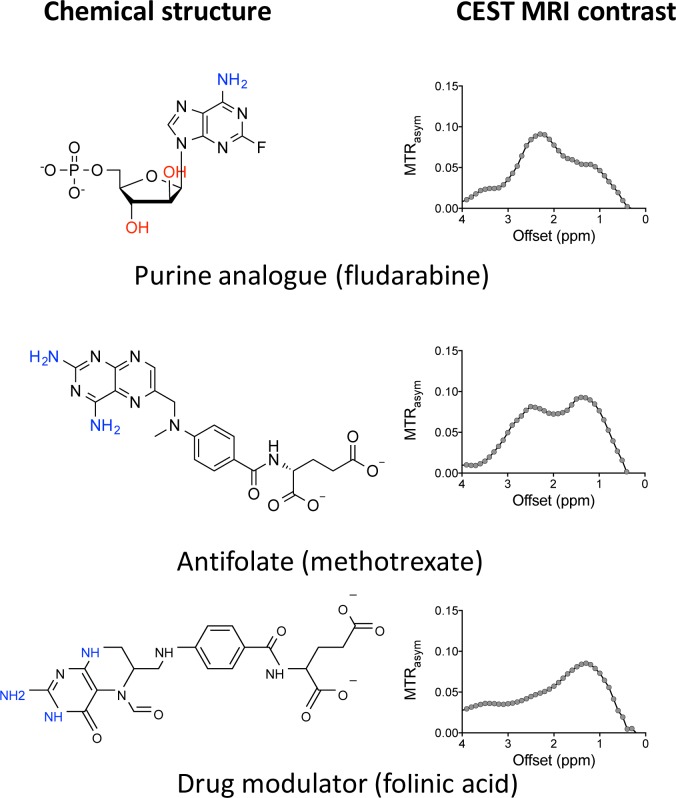
Example of other CEST MRI detectable chemotherapeutic agents All samples were prepared in PBS (pH 7.4) at a concentration of 20 mM and measured at 37°C using a 3.6 μT, 3 sec CW RF pulse.

We then expanded our approach to other antitumor drug categories. Among the twenty-two anticancer drugs (Table S4) that we investigated, antifolates (e.g., methotrexate and the drug modulator folinic acid) and purine analogs (e.g., fludarabine) showed good CEST MRI detectability (Figure 5). These results imply that CEST MRI can be used to detect any drug that has exchangeable protons (amides, amines, and hydroxyls) at the appropriate exchange rate, hence has a widespread application.

Compounds sharing similar chemical structures showed similar CEST signals, indicating the possibility to predict the CEST properties based on the chemical structure of a given drug, as suggested in previous studies [13]. Thus, the properties of exchangeable protons can be substantially affected by surrounding chemical modification, which results in changes in the CEST signal. For example, when pyrimidine is replaced by triazine, the NH_2_ protons of decitabine (Dec) and azacitidine (Aza) show much stronger CEST effects (i.e., MTR_asym_ (2.3 ppm) = 0.23 and 0.31, respectively) than that of dC (i.e., MTR_asym_ (2.1 ppm) = 0.12). In contrast, the CEST signal of hydroxyl protons appears to be mainly affected by the number of protons.

### Use CEST MRI to monitor liposome-mediated drug delivery to the tumor

**Figure 6:**
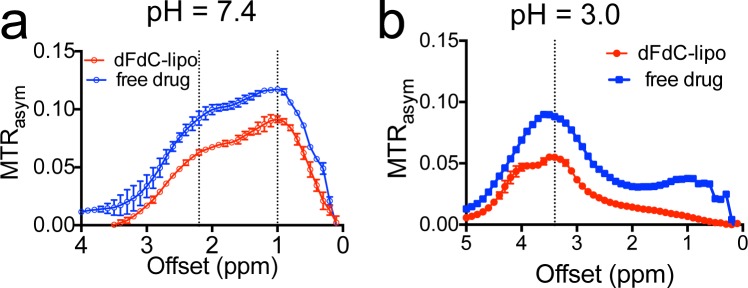
**The CEST of liposomal dFdC (∼80 nM per liposome) and compared with that of free drug (10 mM) at a.** pH 7.4 and **b.** pH 3.0.

The direct visualization of drugs using CEST MRI should allow the label-free tracking of a nanoparticle drug delivery system. To demonstrate this, we encapsulated gemcitabine into liposomes (i.e., liposomal dFdC) and used CEST to monitor the tumor uptake of liposomes in an experimental tumor model. The liposomal dFdC was prepared using a procedure described previously [21, 22] and a liposomal formulation (DPPC: cholesterol: DPPE-PEG = 55:45:5) [23], with an additional 0.5% rhodamine-B-PE (fluorescent dye). The starting solution contained 50 mg/ml gemcitabine hydrochloride (pH ∼ 3). The size of formed liposomes was measured to be ∼120 nm using a dynamic light scattering Nanosizer. The encapsulation rate was estimated as ∼30.8%, using the UV absorbance at 268.8 nm of dFdC. Our *in vitro* drug release assay showed that the initial release of dFdC was very rapid, i.e., ∼34% of loaded dFdC within the first three hours of dialysis. The concentration of intra-liposomal dFdC was then stable and only decreased 4.4% over a period of 24 hours (Figures S4). As shown in Figure 6, the encapsulation of dFdC in liposomes doesn't have a noticeable impact on the CEST properties, as evidenced by the similarity between the shape of the MTR_asym_ plot of liposomal dFdC and that of free form, at both pH 7.4 and pH 3.0.

**Figure 7:**
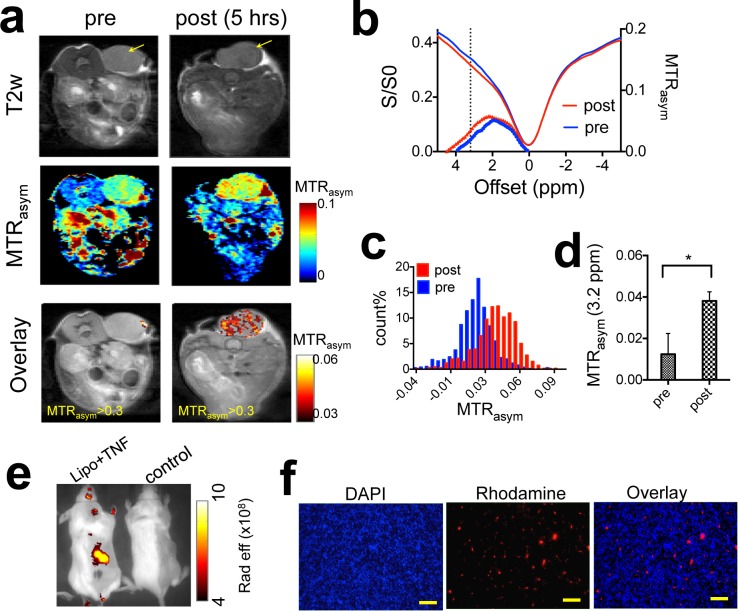
*In vivo* detection of the tumor uptake of liposomal gemcitabine using CEST theranostics **a.** CEST MRI detection of a CT26 tumor (co-treated with TNF-α) before and five hours after the i.v. injection of liposomal dFdC. From top to bottom: T2w images; CEST maps at ∼ 3.2 ppm; and CEST/T2w overlaid images (only the CEST in the tumor region is shown). **b.** Mean tumor CEST signal (MTR_asym_ plots an Z spectra) before and after the injection. **c.** Histogram of the MTR_asym_ values within the tumor regions, before and after the injection of liposomes. **d.** Statistical analysis of mean CEST signal changes in the tumor regions after the injection (*n* = 3). **e.** Whole body fluorescence imaging of a representative mice injected with liposomal dFdC (Gem-lipo) in the presence TNF-α, and a control mouse (saline injection). **f.** Histopathology of tumor section, clearly showing the accumulation and distribution of rhodamine-B-labeled liposomes in the tumor co-injected with liposomes and TNF-α. Nuclei are stained with DAPI (blue). (Scale bar = 100 μm).

We then performed CEST MRI on CT26 tumor-bearing mice before and after the injection of liposomal dFdC. To enhance the intratumoral accumulation of systemically administered liposomes, mice also received a co-treatment of TNF-α, which was shown previously to increase the tumor permeability substantially [24, 25]. Figure 7 shows that liposomal dFdC could be readily detected in TNF-α-treated CT26 tumors five hours after a tail vein injection of 20 mg lipid/kg b.w. (*c.a.* 80 mg dFdC/kg b.w.) and TNF-α (1 μg per mouse). A relatively uniform elevation of CEST MRI signal at 3.2 ppm was conspicuous at five hours after the injection (Figure 7a). The accumulation of liposomal dFdC in the tumor resulted in a net increase of 0.015 in MTR_asym_ (Figure 7b and 7c) as compared to that before the injection. The average increase of CEST contrast in the three tumors studied (Figure 7d) was 0.022±0.012 (the paired two-tailed Student's t test: *P* < 0.05, *n* = 3). The CEST MRI detection of tumor uptake of liposomal dFdC was validated using fluorescence imaging (Figure 7f, S7 & S8). Our results suggest that our approach is capable of directly monitoring delivery of nanoparticulate chemotherapeutics. Moreover, as TNF-α is being investigated clinically for improving the drug delivery of chemotherapy [26], this approach may also be useful for assessing the tumor responses to the combination of nanomedicine and TNF-α or other vascular-targeting treatment.

The capability to directly track drug-loaded nanoparticles without additional imaging labels is expected to be very helpful for both pre-clinical development and the clinical use of nanoparticulate chemotherapeutic agents. There are more than 45 nanoparticulate drug formulations that have been clinically approved, and at least 200 products are currently in Phase I-III clinical trials [27]. If some of them can be tailored into theranostic (therapeutic and diagnostic [28]) systems *via* the proposed CEST MRI method, they can be used directly in the clinic to stratify patients and enable personalized medicine. Moreover, the high local concentration of the drugs loaded in the nanoparticle carriers also helps to boost the sensitivity of CEST, potentially can be used to lessen the requirement of low mM concentration drugs for the CEST MRI detection. Thus our application should have a great impact to nanomedicine in addition to traditional chemotherapies. One limitation of our method is that it is currently unable to detect small molecular drugs at therapeutic concentrations (e.g. ∼ μM). We will continue to work on improving the detectability of the proposed approach with the help of novel CEST techniques [19, 29, 30] in an effort to broaden application. Another technical challenge is the translation of our methods developed on 9.4 T and 11.7T small animal scanners to clinical 3T scanners. While currently many groups are working on this issue and there are indeed a handful of recently developed CEST MRI methods, including APT [12], gluCEST [10], glucoCEST [8, 9], and acidoCEST [31], that have been successfully translated, this will need to be demonstrated for the approach suggested here.

## MATERIALS AND METHODS

### MRI

*In vitro* CEST images were acquired on a 9.4T Bruker Avance system equipped with a 15 mm sawtooth RF coil. A modified RARE sequence (TR = 6.0 sec, effective TE = 43.2 ms, RARE factor = 16, slice thickness = 0.7 mm, FOV = 14x14 mm, matrix size = 128x64, resolution = 0.11x0.22 mm^2^, and NA = 2) [16] including a magnetization transfer (MT) module (one CW pulse, B1 = 3.6 μT (150 Hz), 3 sec) was used to acquire CEST weighted images from −10 ppm to 10 ppm (step = 0.2 ppm) around the water resonance (0 ppm) [32]. The absolute water resonant frequency shift was measured using the WAter Saturation Shift Reference (WASSR) [33] method modified with Lorentzian analysis. The same parameters as in CEST imaging were used except TR = 1.5 sec, t_sat_ = 500 ms, B_1_ = 0.5 μT (21.3 Hz) and the saturation frequency swept from −1 ppm to 1 ppm (step = 0.1 ppm).

*In vivo* images were acquired on an 11.7 T Bruker Biospec horizontal bore scanner (Bruker Biosciences, Billerica, MA) equipped with a 23 mm Circular Polarized MRI transceiver volume coil. The same imaging scheme described above was used with the addition of a fat suppression pulse (3.4 ms hermite pulse, offset = −3.5 ppm). The acquisition parameters were: TR = 5.0 sec, effective TE = 6 ms, RARE factor = 10, t_sat_ = 3 sec, B_1_ = 3.6 μT (150 Hz), slice thickness = 1 mm, acquisition matrix size = 128x64, FOV = 20x20 mm, and NA = 2. Due to the B_0_ field inhomogeneity, we incremented the saturation offset ± 1 ppm (0.1 ppm steps) with respect to water for B_0_ mapping.

Data processing was performed using custom-written scripts in MATLAB (Mathworks, Waltham, MA). CEST spectra were calculated from the mean of an ROI placed over each sample after B_0_ correcting the contrast on a per voxel basis. The CEST signal was quantified using MTR_asym_ at particular offsets of interest (i.e. Δω = +2.2 ppm) using the definition: MTR_asym_ = (S^−Δω^ - S^+Δω^)/ S_0_, where and S^[−Δω +Δω]^ is the water signal intensity in the presence of saturation pulse at offsets ±Δω, and S_0_ is the water signal intensity in the absence of saturation pulses.

### Preparation and characterization of liposomal drugs

DPPC (Avanti Polar Lipids), cholesterol, and DPPE-PEG 2000 (Avanti Polar Lipids) (molar ratios 50:45:5) [23] with an additional 0.5% rhodamine-B-PE (fluorescent dye) were dissolved in chloroform (2 mL). The solvent was removed in vacuum to give a thin lipid film, which was hydrated by shaking in 50 mg/ml gemcitabine hydrochloride (pH ∼ 3) at 50°C for 2 h. The vesicle suspension was sonicated for 30 seconds and then extruded successively through 0.4 and 0.1 μm polycarbonate membranes to obtain the final liposomes with low polydispersity at the desired size. The average size and polydispersity index were then measured by dynamic light scattering experiments on a Zetasizer Nano ZS90 (Malvern Instruments, Southborough, MA). The liposomes were then filtered through Sephadex G-50 gel columns (GE Healthcare Life Sciences, Pittsburg, PA) twice to remove unloaded drugs, and stored at 4°C prior to use. The average size of liposomes was measured as ∼120 nm and final lipid concentration was about 20 mg lipid /ml.

### Animals

All experiments conducted with mice were performed in accordance with protocols approved by the Johns Hopkins University Institutional Animal Care and Use Committee (IACUC). CT26 (CRL-2638) murine colorectal adenocarcinoma cells were purchased from the American Type Culture Collection (ATCC) and grown in McCoy's 5A Medium (Invitrogen/Life Technologies, Carlsbad, CA) supplemented with 10% Fetal Bovine Serum (FBS, HyClone, Thermo Scientific, Waltham, MA) at 37°C with 5% CO_2_. Five million CT26 cells were injected subcutaneously into the right flank of female BALB/c mice (6-8 weeks; Harlan, Indianapolis, IN; ∼20 g in weight), and allowed to grow for ∼10 days. Ten days after implantation, mice (n = 3, each group) received a tail vein injection of 100 mg lipid/kg b.w. (c.a. 80 mg gemcitabine/kg b.w.) with or without TNF-α (1 μg per mouse). TNF-α was reconstituted freshly before administration in doubly- distilled H_2_O at 100 μg/mL and diluted into 0.1% (w/v) BSA in PBS at a final concentration of 10 μg/mL. Liposomal gemcitabine was injected within a few minutes thereafter. The CEST images were acquired at 4-5 hours after the injection.

### Fluorescence imaging

Fluorescence imaging was performed and analyzed using a Spectrum/ CT IVIS^®^ *in vivo* imaging system with the Living Image^®^ software (PerkinElmer, Waltham, MA). Fluorescence signal (emission = 620 nm, excitation = 570 nm) was quantified as radiant efficiency.

### Immunohistochemistry

Excised tumors were imaged immediately after MRI measurements and processed for histology. Tumor sections of 10 μm were stained with 4′,6-diamidino-2-phenylindole (DAPI) for nuclei and examined under an inverted microscope (Olympus, Tokyo, Japan) for DPAI (blue) and rhodamine-B conjugated with liposomes (red).

### Statistics

All *in vitro* experiments reported were performed in triplicate. Quantitative data are expressed as mean ± SD, as indicated. Statistical significance was assessed by the two-tailed unpaired Student's *t*-test. Values of *P* < 0.05 were considered significant and asterisked.

## CONCLUSIONS

In summary, we have demonstrated a label-free imaging approach to “see” drugs directly, namely CEST theranostics. We screened a wide array of chemotherapeutic agents *in vitro* and confirmed the CEST MRI contrast of the drugs and their nontoxic analogs in three major categories: pyrimidine analogs, purine analogs, and antifolates. We also showed that CEST MRI could be used synergistically with nanomedicine to transform currently available therapeutics directly into theranostics, which enabled the first successful CEST MRI detection of the tumor uptake of liposomal gemcitabine without need for synthetic imaging labels. These results imply that we can potentially transform many currently available drugs, including those already in the clinic and those still under pre-clinical development, to be MRI-detectable theranostic agents, WITHOUT any radioactive-, paramagnetic-, or super-paramagnetic-based labeling.

## SUPPLEMENTARY MATERIAL FIGURES AND TABLES


