# Supplementary material for: CEST theranostics: label-free MR imaging of anticancer drugs
Source: Oncotarget. 2016 Feb 2;7(6):6369–78. doi: 10.18632/oncotarget.7141 (PMC4872720; doi:10.18632/oncotarget.7141)
Supplement: Supplementary file 1 [file oncotarget-07-6369-s001.pdf]

CEST theranostics: label-free MR imaging of anticancer drugs

Supplementary Material

S1. The effect of saturation parameters on the CEST contrast of cytidine based anticancer drugs

To determine the optimal saturation pulse, we acquired the Z-spectra and MTR_{asym} spectra of the cytidine based anticancer drugs, gemcitabine (dFdC), cytarabine (araC), decitabine (Dec), and azacitidine (Aza) and their natural analog deoxycytidine (dC) using, 1) varied B_1: 1.2, 2.4, 3.6, 4.7 and 5.9 μT with a fixed T_{sat}= 4 seconds; or 2) varied pulse duration (T_{sat}) of 0.5, 1, 1.5, 2.5, 4, 6 seconds with a fixed B_1= 4.7 μT. All samples were prepared at a concentration of 20 mM and pH 7.4. Triplicate samples were prepared at each condition for each agent. The results are shown in Figure S1.

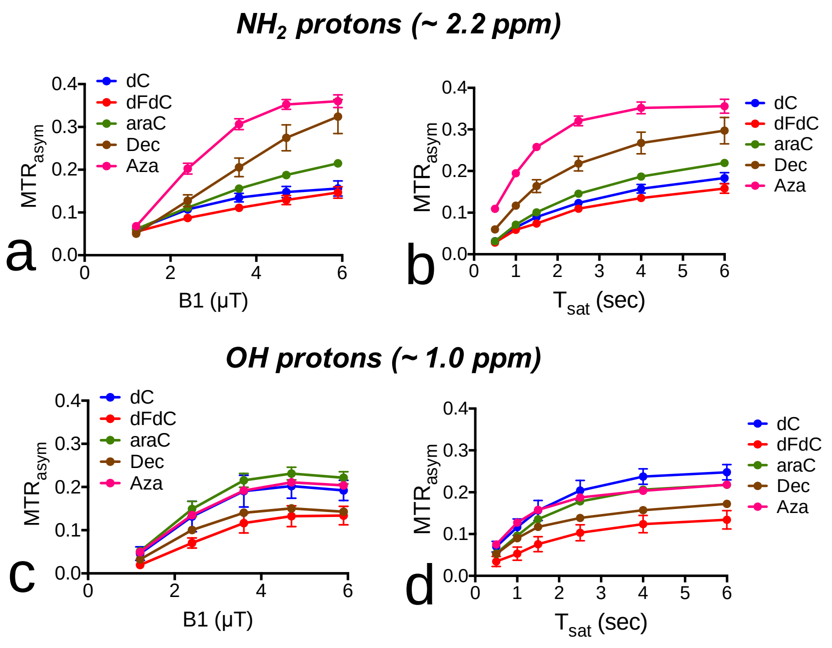

Figure S1. The effect of the power (B_1) and duration of saturation pulse on the CEST MRI detection of NH_2 protons (~ 2.2 ppm, **a** and **b**) and hydroxyl protons (~ 1.0 ppm, **c**

and **d**) cytidine-based anticancer drugs. All samples were prepared in PBS (pH 7.4) at a concentration of 20 mM and measured at 37 ^{o}C). The results are mean ± STD (n=3). Note that the offset frequencies of hydroxyl protons were found to differ slightly between different agents. For the plots shown in **c** and **d**, we used 1.2 ppm for Aza and Dec.

These results can be used to estimate the practically useful RF pulse parameters for acquiring maximum CEST MRI. For example, the good saturation parameters for detecting dFdC and dC, are B_1=3.6 μT and T_{sat}= 3 seconds. Further increasing saturation power and length won't increase the CEST contrast much. Instead it will increase water direct saturation and competing semi-solid magnetization transfer effects, as well as the SAR.

S2. The pH dependence of gemcitabine and other cytidine-based anticancer drugs

The pH dependence of other cytidine-based anticancer drugs between pH 6.5 and pH 8 was measured and the results are shown in Figure S2.

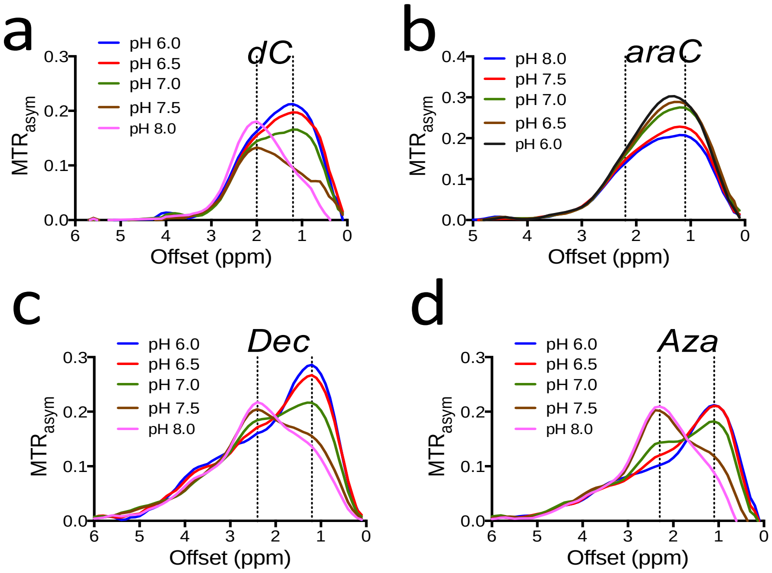

Figure S2. The pH dependent CEST contrast of several cytidine analogues: a) dC, b) araC, c) Dec and d) Aza.

We also used the frequency-labeled exchange (FLEX) transfer method[1] as previously described[2] to determine the exchange rate of amino protons for each drug. In brief, FLEX experiments were conducted at 17.6 T on a Bruker Avance III spectrometer using a micro2.5 microimaging probe. Each FLEX acquisition consisted of 600 LTMs, each LTM containing excitation pulses with flip angle = 90 degrees, duration = 0.15 ms, and the carrier frequency was placed 8.6 ppm downfield from the water resonance. The t_{exch} was set to 8 ms for a total preparation time of 5 s. The FLEX labeling period was followed by a single-shot fast spin-echo (FSE) imaging readout with TR/TE =

15 s/7.8 ms. In total, 100 images were acquired with t_{evol} ranging from 0 ms to 3 ms. FLEX data was processed using a collection of python packages (www.numpy.org, www.scipy.org, and www.matplotlib.org). The estimated exchange rates of the amino protons on each compound are listed in Table S1. It should be noted that due to the small difference in the chemical shifts of NH_2 and OH, the OH protons could have a strong effect on the measurement of NH_2 protons.

Table S1 Chemical shift, exchange rate and pKa of NH_2 protons

	**dC**	**dFdC**	**araC**	**Dec**	**Aza**
**Chemical shift (ppm)**	2.0	2.2	2.1	2.4	2.3
**k_{ex} (Hz)**	1007	812	1140	484	623
**pKa of NH_2***	4.3	4.3	4.3	3.3	3.5

*Calculated using Advanced Chemistry Development (ACD/Labs) Software V11.02

We also used the same FLEX method to determine the exchange rate of dFdC at different pH. As shown in Table S2, the change in exchange rate is consistent with the trend of CEST contrast change shown in Figure S2c.

Table S2 Exchange rate of the NH_2 protons of dFdC at different pHs

	**pH 6.0**	**pH 6.5**	**pH 7.0**	**pH 7.4**	**pH 8.0**
**k_{ex} (Hz)**	952	537	602	812	951

S3. Definition of CNR in the CEST MRI detection In the current study, the CNR (between pre- and post- contrast) was determined using the equation below[3],

$$CNR = (SNR^{-\Delta\omega} - SNR^{+\Delta\omega}) = \frac{S^{-\Delta\omega} - S^{+\Delta\omega}}{noise} = \frac{S^{-\Delta\omega} - S^{+\Delta\omega}}{S_0} \cdot \frac{S_0}{noise} = MTR_{asym} \cdot SNR^{S0}$$

Where SNR is the signal-to-noise Ratio and SNR^{S0} is the SNR determined using the S_0 image (no saturation). Noise was estimated from the standard deviation (δ) of background noise in the S_0 image. $S^{+\Delta\omega}$ and $S^{-\Delta\omega}$ are the MRI signal intensities after saturation at

negative and positive values of the offset frequency $\Delta\omega$ from the water proton frequency (which is set at 0 ppm by convention).

The difference in CEST contrast between a gemcitabine-containing sample and its corresponding reference sample then was defined as ΔCNR,

$$\Delta CNR = CNR^{agent} - CNR^{control}$$

where CNR^{agent} is the CNR of a agent-containing samples and $CNR^{control}$ is the CNR of a reference sample (either PBS or gel).

S.4. Comparison of CEST and ^{19}F MRI measurements on detecting dFdC

Because dFdC contains two ^{19}F atoms in its structure, it can also be detected by ^{19}F MRI[4]. We therefore performed a comparative study of CEST MRI and ^{19}F MRI on the same set of phantoms. The result is shown in Figure S5.

MRI experiments were performed on a vertical 17.6 T scanner (Bruker Avance system) at 37°C. A 15 mm birdcage radiofrequency coil was used to acquire both ^{1}H and ^{19}F MR images by sweeping the coil frequency from proton (750 MHz) to fluorine (705 MHz) frequency. For *^{1}H MRI CEST*: a slightly modified CEST MRI method as that described in *section S1* was used with the following parameters: TR/TE=10,000/46 ms, RARE factor=32, t_{sat}=4 sec, B_1=3.6 µT (150 Hz), 5 mm slice thickness, FOV=1.4×1.4 cm, matrix size=64×32, spatial resolution = 220x440 µm, and single average (NA=1); for *^{19}F MRI*: a modified RARE sequence (TR=1,000 ms, effective TE=6ms, RARE factor=8, centric encoding) with the exact geometry as the CEST MRI. The number of averages for ^{19}F MRI was set to 128 so that the total acquisition was 8 min 32 sec, which was approximately the same as that of spectral CEST MRI acquisition (51 offsets, 8 min

30 sec). In order to make the acquisition times of the two MRI measurements comparable, we didn't acquire any WASSR spectra. Instead, we used the Z-spectral data to correct the B_0 inhomogeneity on a pixel by pixel basis. This approach worked for our phantom study because these samples (in PBS solutions) don't have interfering semi-solid MT effects and only have CEST effects on the positive side of Z-spectra.

It should be noted that, in practice, there is no need to always collect a full Z-spectrum to determine the CEST effect at a particular offset frequency. To estimate the T_2 relaxation times of ^{19}F atoms of dFdC, a Multi Slice Multi Echo (MSME) sequence was performed with the following parameters (matrix=32*32, number of slices=1, slice thickness=5 mm, TR=5000 ms, TE=6 ms, number of echos=20; NA=128). The T_2 relaxation time was estimated to be 11 ms.

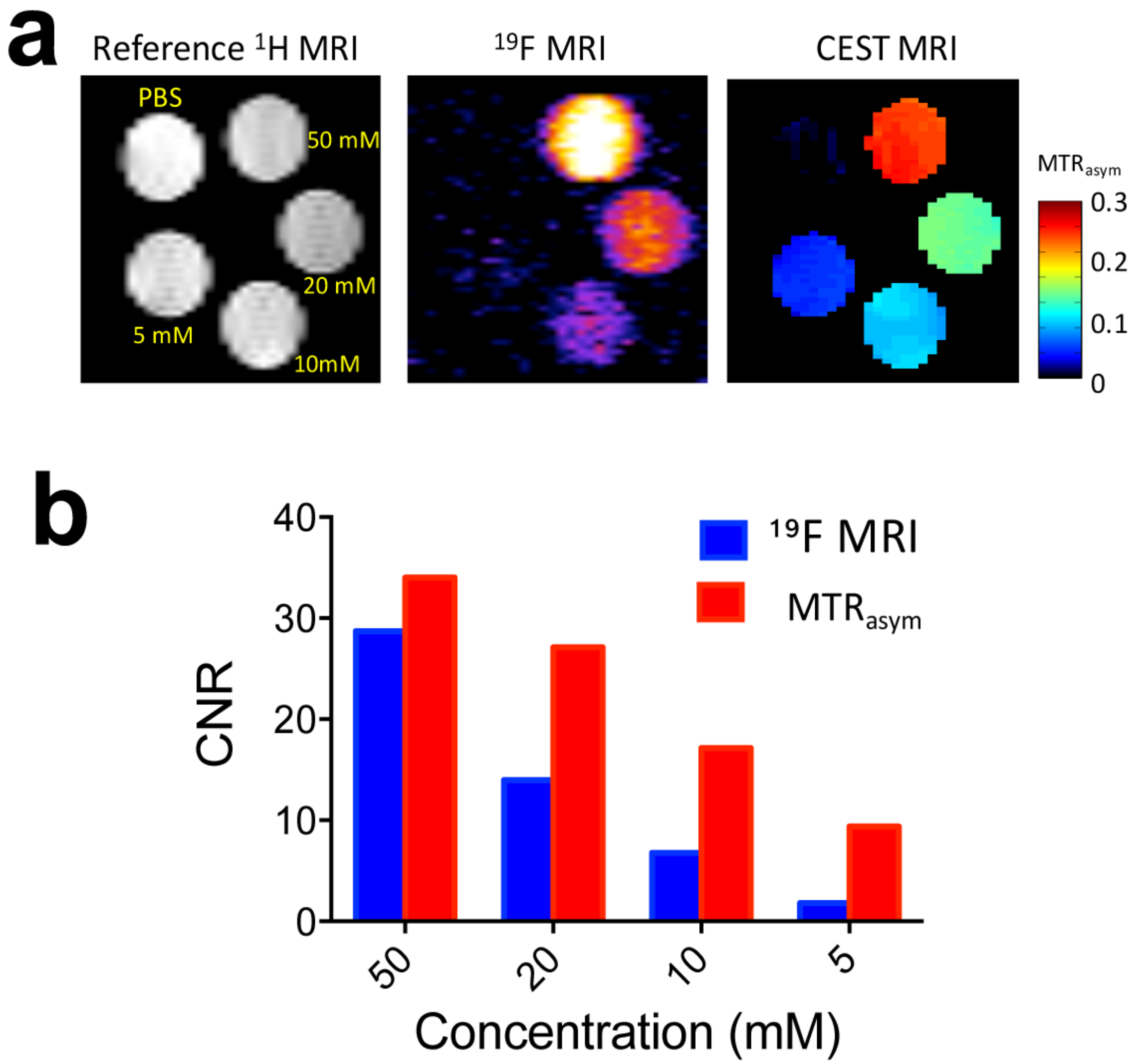

Figure S3. Comparison of the detectability of CEST MRI and ^{19}F MRI at 17.6 Tesla MRI scanner. **a:** from left to right: T2-weighted (reference 1H MRI image (T2-weighted image), ^{19}F MRI image and the MTR_{asym} parametric map of five samples containing gemcitabine at concentrations of 0, 5, 10, 20, and 50 mM. **b:** The comparison of contrast-to-noise ratio (CNR) of ^{19}F MRI and CEST MRI at each concentration, as opposed to the signal of PBS. CNR is calculated by comparing the difference between the ^{19}F MRI signal or CEST contrast in a dFdC sample and those in PBS, normalized by the noise.

S.5. Screening of CEST detectable anticancer drugs

In addition to the cytidine-based anticancer drugs, we also screened 17 other compounds including anticancer drugs that are either approved or in clinical trials and their natural analogs. To select the potential drugs with CEST signal, we first searched for several prototype drugs from the online drug database DrugBank (www.drugbank.ca), which contains detailed information of 6811 drug entries including 1528 FDA-approved small molecule drugs, 150 FDA-approved biotech (protein/peptide) drugs, 87 nutraceuticals and 5080 experimental drugs. The criteria for the search were 1) drugs that share a chemical component or structure similar to that of a known compound with detectable CEST contrast (criterion 1a); or other drugs whose exchangeable protons are likely to be in the slow-to-moderate range based on previous reports or experiences (criterion 1b); 2) drugs with a maximum tolerated dose (MTD) > 300 mg/m^2 (Medline search); 3) drugs that are widely used in the clinic. It should be noted that 300 mg/m^2 corresponds to 8 or 100 mg/kg for human or mouse respectively. It is because that the appropriate way to convert drug doses from animal studies to human studies, or vice versa, is to use the body surface area (BSA) normalization method. Converting doses from mg/m^2 to mg/kg is defined by dose(mg/m^2)=dose(mg/kg) x km, where km is the Representative Surface Area to Weight Ratio, which is 37.0 and 3.0 for human and mouse respectively[5, 6].

The selected drugs were screened using a high throughput screening CEST MRI method, which allows a simultaneous screen of up to 46 samples (26 samples in the original publication[3]). Selected drugs were evaluated for their pH dependence (6.5-8.0), and optimized with respect to B_1 strength (1.2-5.9 μT).

We first evaluated several natural analogs of anticancer drugs. The CEST plots for these compounds (pH =7.4) are shown in Table S3, indicating that all of them have favorable CEST signals.

Table S3 The chemical structure and CEST contrast for selected natural compounds

**Anticancer drug Category**	**Natural analogs**	**CEST MRI**
**antifolate**	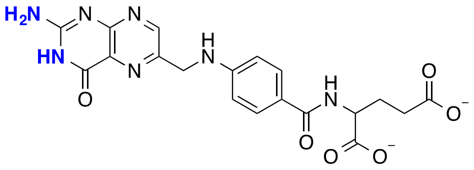 folic Acid (vitamin B9)	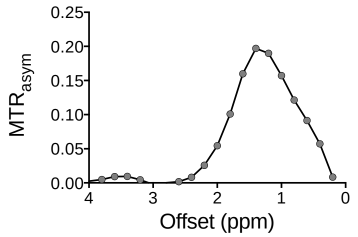
**pyrimidine analogs**	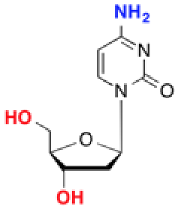 deoxycytidine	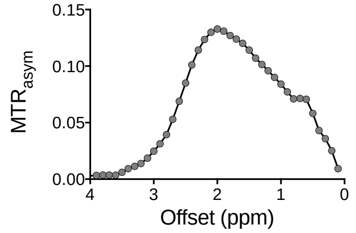
**purine analogs**	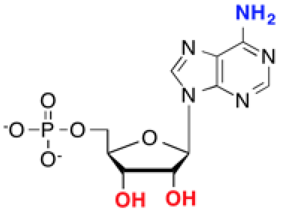 AMP	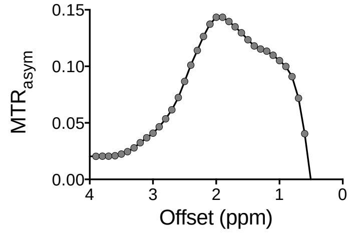

The CEST MRI plots of other 13 anticancer drugs (20 mM unless otherwise noted), in addition to those shown in the main text, including both CEST detectable and undetectable are collectively shown in Table S4.

Table S4. Chemotherapeutic agents screened in current study

Anticancer drugs	Chemical structure	CEST contrast
2'-deoxyinosine	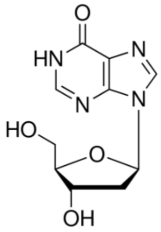	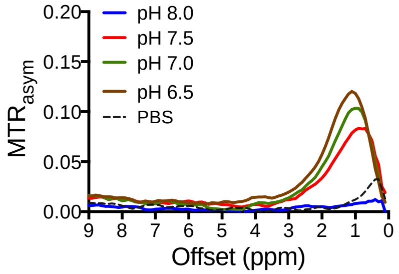
fludarabine	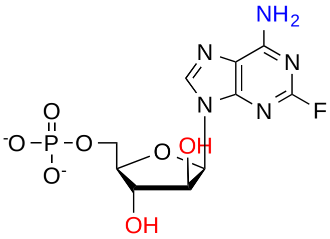	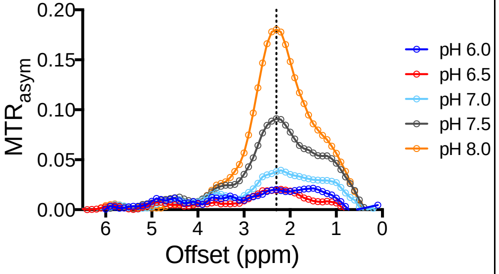
cladribine (5 mM, only pH 7.4 was measured)	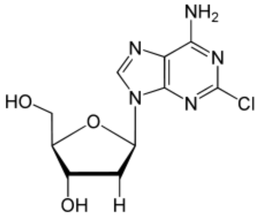	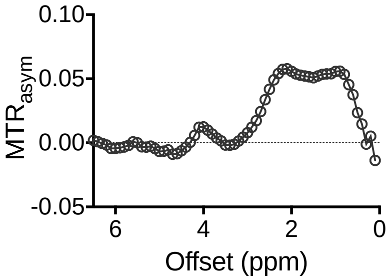
clofarabine	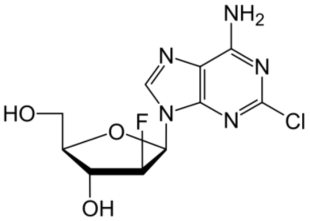	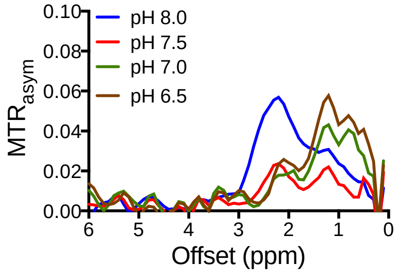

5-fluorodeoxyuridine monophosphate, 5-FdUMP (10 mM)	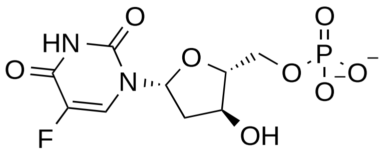	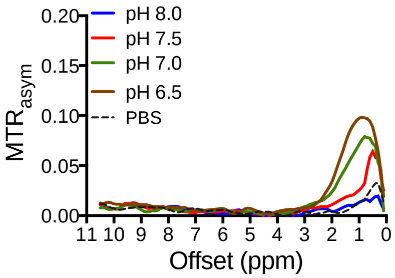
folinic acid (10 mM)	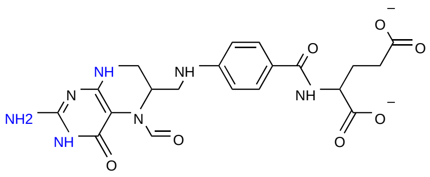	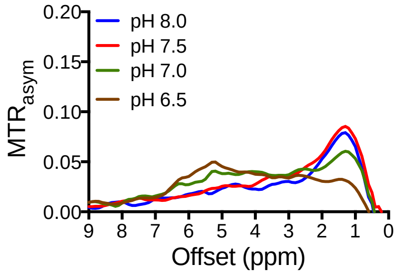
pemetrexed	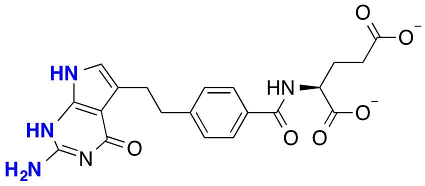	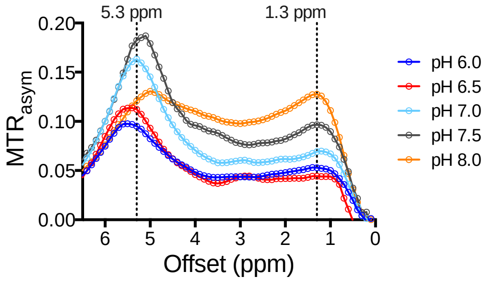
neomycin (10 mM)	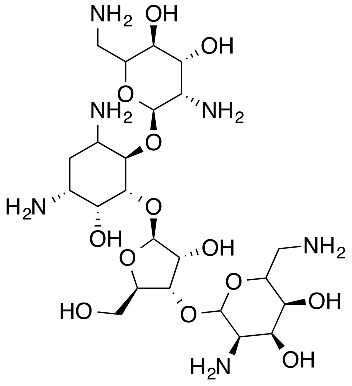	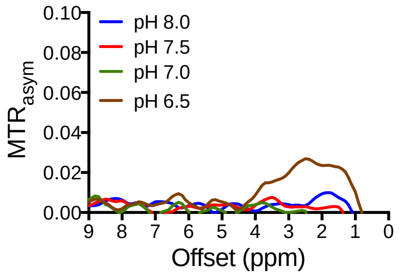

cimetidine	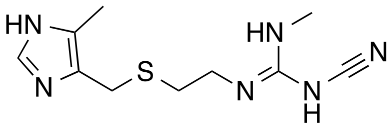	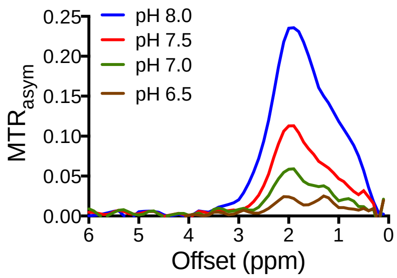
mitoxantrone	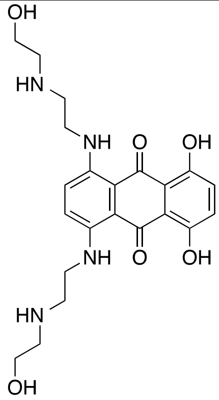	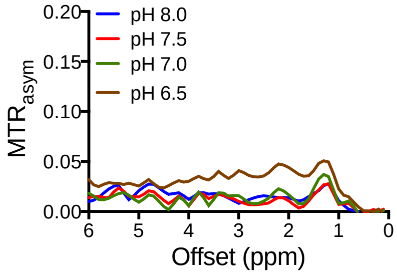
mitomycin C	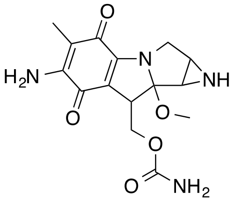	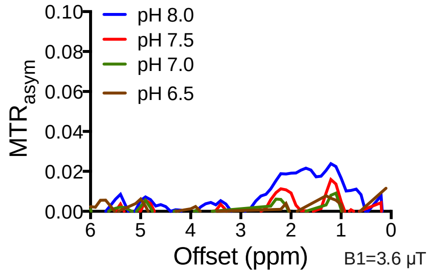
carboplatin	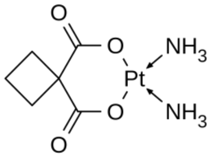	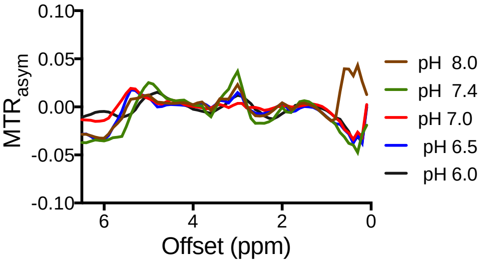

cisplatin (10 mM)	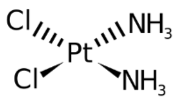	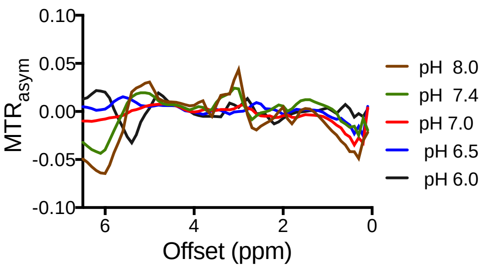

S6. Characterization of liposomal drugs To determine the stability of the prepared liposomes, we used both CEST and UV absorbance (OD268.8) to assess the retained dFdC in liposome over time. In brief, liposomes were filtered immediately after formation using a G50 column to remove unencapsulated drug. Six samples of 0.3 ml liposome solution were placed in a 0.5 ml dialysis cassette (3.5 KD MW cut off, Thermo scientific), and each of them was immersed in 40 mL PBS buffer under continuous stirring, which was sufficient to produce a good sink condition considering that the solubility of gemcitabine in PBS is approximately 2 mg/ml. At each time point, i.e., 0, 1, 2, 3, 4, and 24 hours, one sample was removed for measurement of CEST contrast. After the CEST MRI, the sample was suspended in a 10% v/v Triton X-100 solution and thoroughly agitated using a water bath sonicator at 42 °C, followed by centrifugation (21,000 ×g, 10 min), followed by UV absorbance measurement at 268.8 nm using a UV-Vis spectrophotometer[7].

To determine the stability in serum, filtered liposomes were mixed with an equal volume of fetal bovine serum (FBS) to a final volume of 450 µl in a dialysis cassette (3.5 KD MW cut off)[8]. The dialysis cassette then was immersed in 60 mL PBS. At each time point, i.e., 0, 1, 2, 3, 4, and 24 hours, a 3 mL sample of dialysis solution was taken

out to measure the OD268 to quantify the released drug in the dialysis solution. Quickly after the measurement, the sample was put back in so that the volume of the dialysis solution was kept the same throughout the experiment. The initial concentration of gemcitabine in liposomes was determined by measuring the OD268 of one liposome sample right after G50 filtering by suspending in a 10% v/v Triton X-100 solution and agitation using a water bath sonicator at 42 °C.

The results are shown in Figure S4. A burst release is observed within the first 4 hours. After then, the release rate of dFdC from liposome is slow and >60% of loaded dFdC could be retained in 24 hours. There is a good consistency between the UV measurement and CEST MRI. The release rate in the presence of serum is markedly faster. However still 44.6% of encapsulated gemcitabine could be retained in the lumen of liposomes within the first 24 hours.

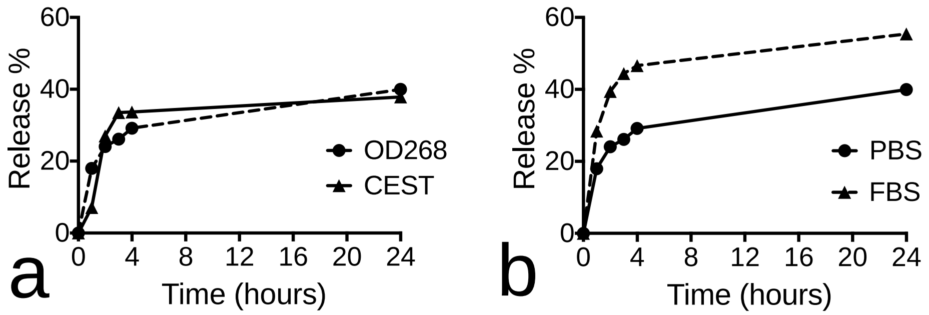

Figure S4. The release profile of dFdC from liposomes: a) the release profile measured by OD268 (dashed line) and CEST MRI (solid line, quantified by MTR_{asym} at 2.2 ppm); and b) in the absence of serum (solid line) or in the presence of FBS (dashed line).

S7. *In vivo* MRI results

The results for all the animals are shown in Figure S5 and Figure S6.

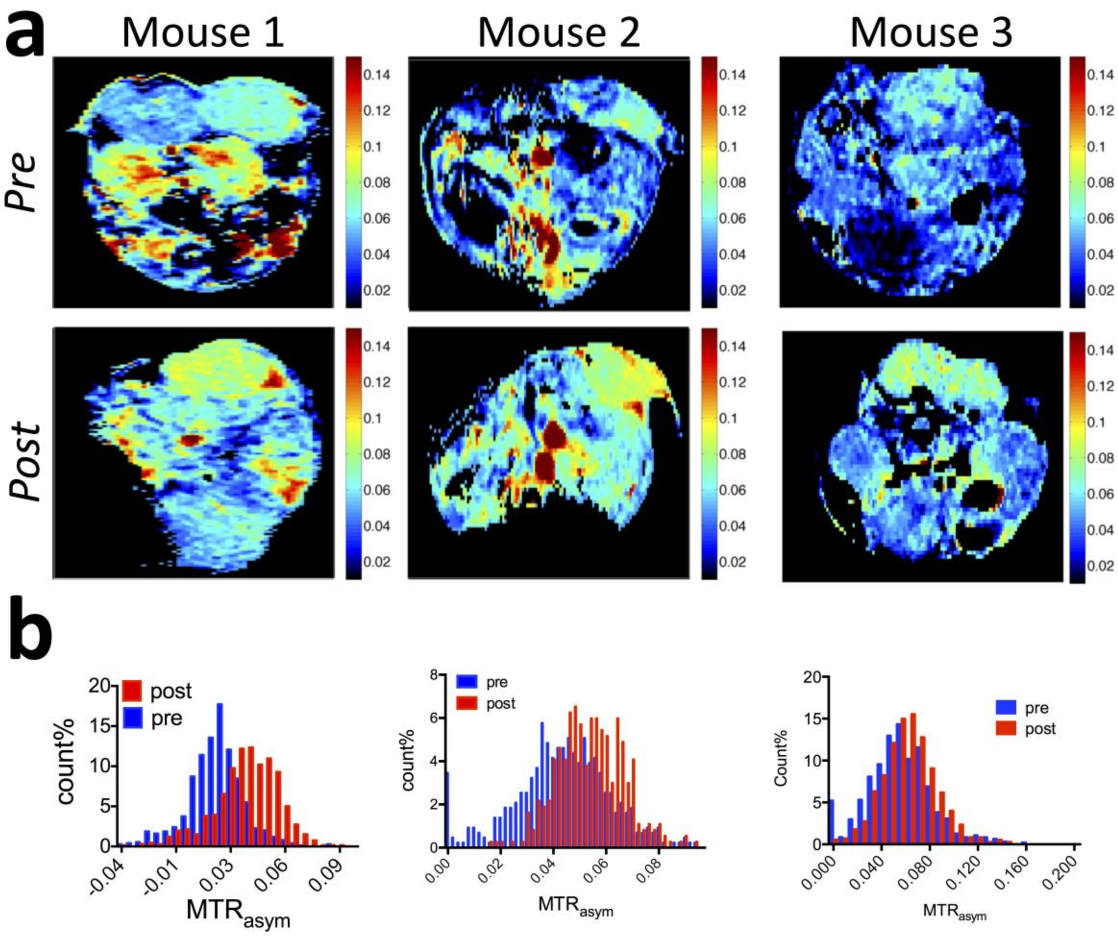

Figure S5. CEST images of three mice co-injected with liposomal dFdC and TNF-α. **a)** The CEST maps (MTR_{asym} at 3.2 ppm) before (top) and 4-5 hours after (bottom) of the injection. **b)** Histogram of the MTR_{asym} values within the tumor regions, before and after the injection of liposomes.

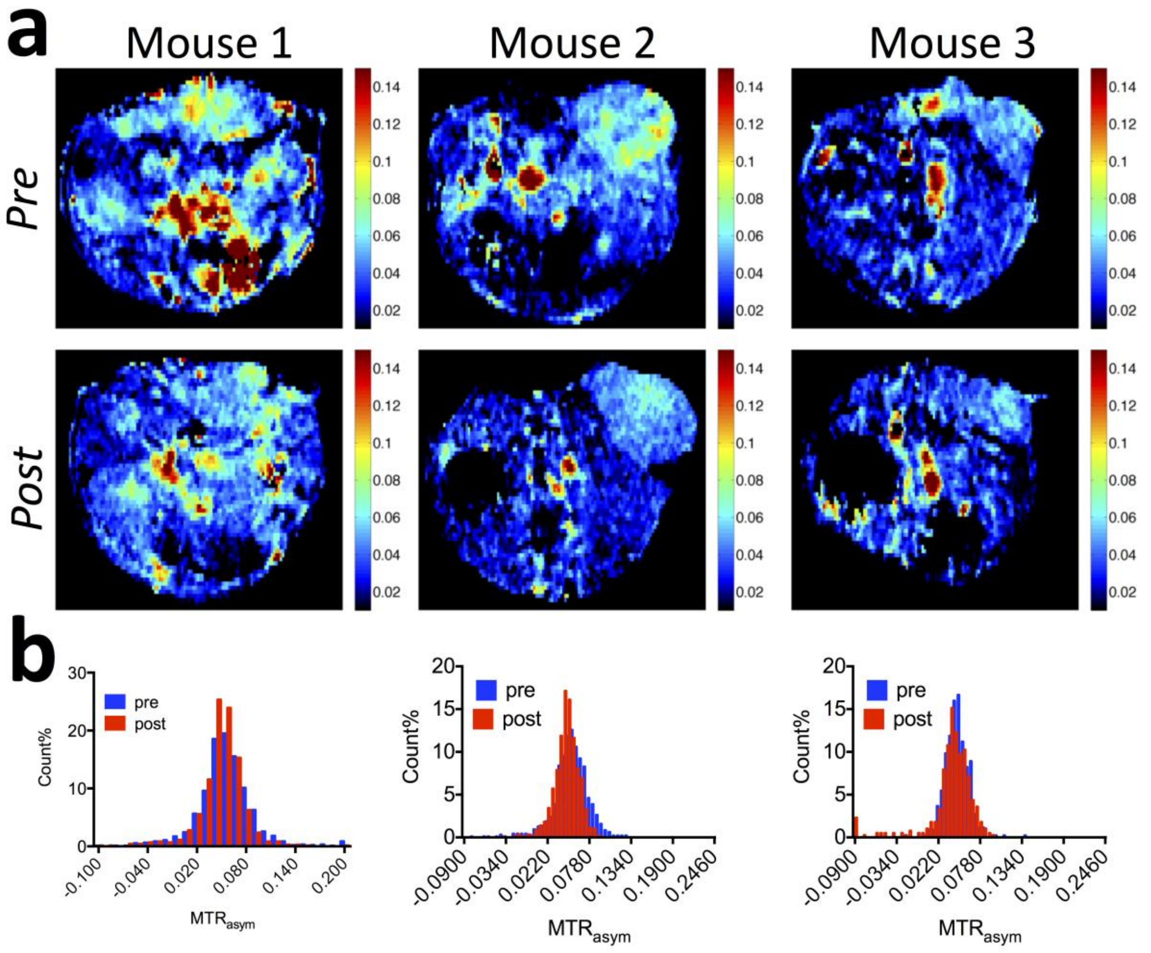

Figure S6. CEST images of three mice injected with liposomal dFdC only. **A:** The CEST maps (MTR$_{asym}$ at 3.2 ppm) before (top) and 4-5 hours after (bottom) of the injection. **b**: Histogram of the MTR$_{asym}$ values within the tumor regions, before and after the injection of liposomes.

S8. Validation

In vivo fluorescence imaging of the tumor uptake of liposomes

Fluorescence imaging was performed and analyzed using a Spectrum/ CT IVIS® in vivo imaging system with the Living Image® software (PerkinElmer, Waltham, MA).

Fluorescence signal (emission = 620 nm, excitation = 570 nm) was quantified as radiant efficiency. The exposure time for each image acquisition was 1 s. Images were scaled to the same maximum intensity using the supplier's software. Because rhodamine-B-PE is incorporated into the liposome bilayer, the fluorescent signal is an excellent representation of the location and amount of liposomes[9, 10].

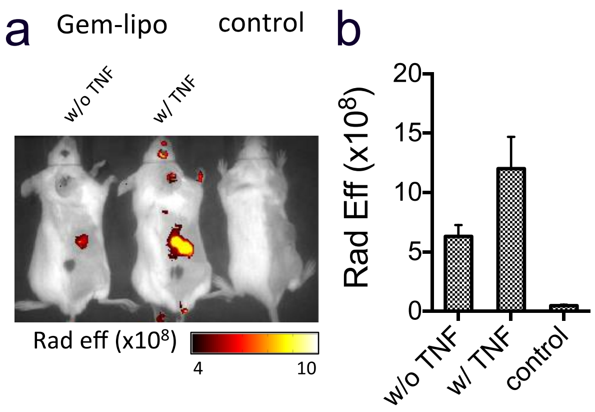

Figure S7. Whole body fluorescence imaging of two representative mice injected with liposomal dFdC (Gem-lipo) in the presence or absence of TNF, and a control mouse without any injection and the mean fluorescence intensity of the ROIs over the tumor regions.

<u>Biodistribution assessed by ex vivo fluorescence</u>

Immediately after MRI measurements and in vivo fluorescence imaging, mice were sacrificed by cervical dislocation and tumor, brain, liver, spleen, kidneys, and lung were collected and assembled on a Petri dish for image acquisition. All images were scaled to the same maximum intensity for direct comparison. For quantification, regions of interest (ROI) were drawn over the organs displayed in ex vivo images (n = 3) and fluorescence signal intensity of the organs was calculated using the supplier's software.

<u>Immunohistochemistry</u>

Excised tumors were imaged immediately after MRI measurements and processed for histology. Tumor sections of 10 μm were stained with 4′,6-diamidino-2-phenylindole (DAPI) for nuclei and examined under an inverted microscope (Olympus, Tokyo, Japan) for DPAI (blue) and rhodamine conjugated with liposomes (red).

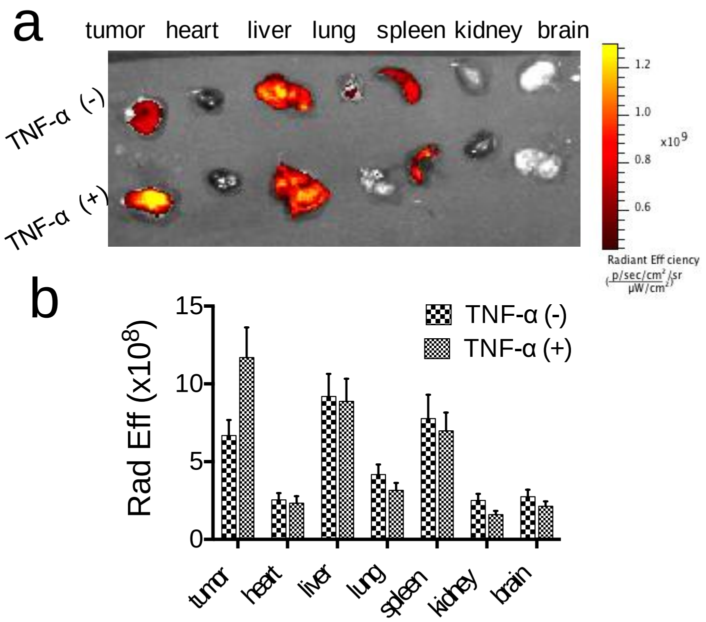

Figure S8. The biodistribution of liposomes at 5 hours after the injection using the fluorescence signal of the liposomes. **a)** The ex vivo organ imaging; **b)** Biodistribution data from regions of interest (ROI) drawn over organs displayed in ex vivo images.

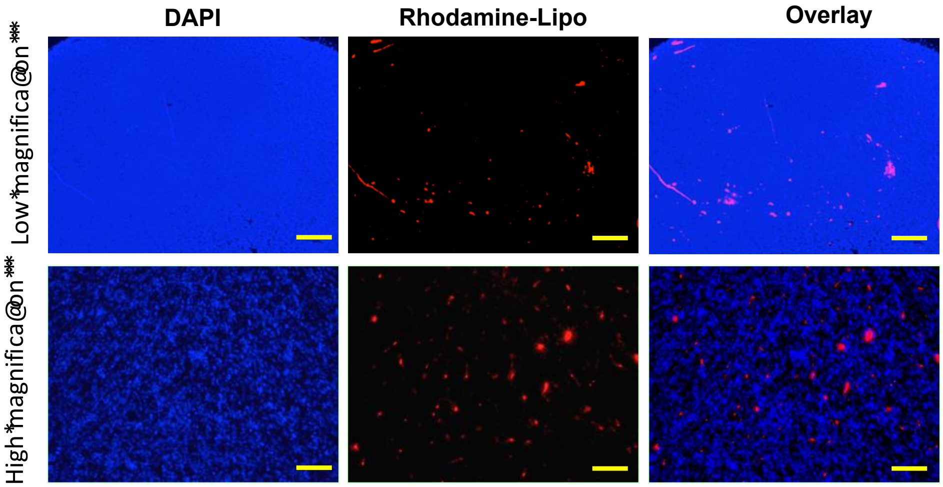

Figure S9. Immunohistochemistry of tumor sections, clearly showing the accumulation and distribution of rhodamine-labeled liposomes in the tumor co-injected with liposomes and TNF-α. Nuclei are stained with DAPI (blue). (Scale bar = 500 and 100 μm for the top panel and bottom panel respectively).

References

1. Friedman JI, McMahon MT, Stivers JT and Van Zijl PC. Indirect detection of labile solute proton spectra via the water signal using frequency-labeled exchange (FLEX) transfer. J Am Chem Soc. 2010; 132(6):1813-1815.

2. Liu G, Liang Y, Bar-Shir A, Chan KW, Galpoththawela CS, Bernard SM, Tse T, Yadav NN, Walczak P, McMahon MT, Bulte JW, van Zijl PC and Gilad AA. Monitoring enzyme activity using a diamagnetic chemical exchange saturation transfer magnetic resonance imaging contrast agent. J Am Chem Soc. 2011; 133(41):16326-16329.

3. Liu G, Gilad AA, Bulte JW, van Zijl PC and McMahon MT. High-throughput screening of chemical exchange saturation transfer MR contrast agents. Contrast Media Mol Imaging. 2010; 5(3):162-170.

4. Cron GO, Beghein N, Ansiaux R, Martinive P, Feron O and Gallez B. 19F NMR in vivo spectroscopy reflects the effectiveness of perfusion-enhancing vascular modifiers for improving gemcitabine chemotherapy. Magn Reson Med. 2008; 59(1):19-27.

5. Freireich EJ, Gehan EA, Rall DP, Schmidt LH and Skipper HE. Quantitative comparison of toxicity of anticancer agents in mouse, rat, hamster, dog, monkey, and man. Cancer Chemother Rep. 1966; 50(4):219-244.

6. Reagan-Shaw S, Nihal M and Ahmad N. Dose translation from animal to human studies revisited. FASEB J. 2008; 22(3):659-661.

7. Grazia Calvagno M, Celia C, Paolino D, Cosco D, Iannone M, Castelli F, Doldo P and Fresta M. Effects of lipid composition and preparation conditions on physical-chemical properties, technological parameters and in vitro biological activity of gemcitabine-loaded liposomes. Curr Drug Deliv. 2007; 4(1):89-101.

8. MAGIN RL and NIESMAN MR. Temperature-dependent drug release from large unilamellar liposomes. Cancer Drug Deliv. 1984; 1(2):109-117.

9. Campbell RB, Fukumura D, Brown EB, Mazzola LM, Izumi Y, Jain RK, Torchilin VP and Munn LL. Cationic charge determines the distribution of liposomes between the vascular and extravascular compartments of tumors. Cancer Res. 2002; 62(23):6831-6836.

10. Kluza E, Jacobs I, Hectors SJ, Mayo KH, Griffioen AW, Strijkers GJ and Nicolay K. Dual-targeting of αvβ3 and galectin-1 improves the specificity of paramagnetic/fluorescent liposomes to tumor endothelium in vivo. J Control Release. 2012; 158(2):207-214.
